# Microbial Metabolomes in Alzheimer’s Disease: From Pathogenesis to Therapeutic Potential

**DOI:** 10.3390/cimb47090724

**Published:** 2025-09-05

**Authors:** Alejandro Borrego-Ruiz, Juan J. Borrego

**Affiliations:** 1 Departamento de Psicología Social y de las Organizaciones, Universidad Nacional de Educación a Distancia (UNED), 28040 Madrid, Spain; 2 Departamento de Microbiología, Universidad de Málaga, 29071 Málaga, Spain

**Keywords:** Alzheimer’s disease, gut microbiome, microbiota-gut–brain axis, microbial metabolomes, therapeutic approaches

## Abstract

Background: Accumulating evidence underscores the potential role of the gut microbiome in the pathogenesis of Alzheimer’s disease, but much remains to be clarified. This review examines current evidence linking gut microbiome dysbiosis to Alzheimer’s disease, focusing on microbial metabolomes and their mechanistic role, as well as on the potential of therapeutic approaches targeting the gut microbiome. Methods: A narrative, non-systematic examination of the literature was conducted to provide a comprehensive overview of the subject under examination. Database searches were performed in PubMed, Scopus, and Web of Science between June and July 2025. Results: Alzheimer’s disease is linked to reduced gut microbial diversity and altered bacterial taxa. Gut microbiome shifts correlate with inflammation and may drive Alzheimer’s disease progression via the microbiota–gut–brain axis. Microbial amyloids and bacterial products can cross both the intestinal and blood–brain barrier, triggering neuroinflammation and promoting amyloid and tau pathologies. Short-chain fatty acids produced by the gut microbiome regulate neuroinflammation, lipid metabolism, and gene expression, impacting Alzheimer’s disease pathology. Therapeutics targeting the gut microbiome, including probiotics, prebiotics, and fecal microbiota transplantation, show promise in modulating neuroinflammation, reducing amyloid and tau pathology, and improving cognitive function in Alzheimer’s disease. Conclusions: The gut microbiome significantly influences Alzheimer’s disease pathogenesis, and its modulation offers potential to slow progression. However, further research is required to validate effective clinical interventions.

## 1. Introduction

Alzheimer’s disease (AD) is a progressive neurodegenerative condition characterized by cognitive deterioration, including memory loss, recognition impairments, and executive dysfunction [1]. The World Health Organization (WHO) estimated that, in 2021, 57 million individuals worldwide were living with dementia, with AD accounting for 60–70% of cases, thus representing the most common form of dementia [2]. Age is recognized as the most significant risk factor for AD and other forms of dementia, with risk rising sharply after the age of 60. While global epidemiological shifts have had only a minor overall impact, substantial variation exists across regions and between sexes. Bayesian age-period-cohort modeling predicts sustained growth through 2040, with age-standardized incidence and prevalence rates projected to reach 144.85 and 821.80 per 100,000, respectively. This increase is driven primarily by population aging in high socio-demographic index (SDI) regions and by demographic expansion in low-SDI regions [3]. Epidemiological projections estimate that the affected population will rise to 78 million by 2030 and reach 139 million by 2050 [2], posing a substantial challenge to the long-term viability of healthcare systems across the world and potentially leading to their collapse if adequate preventive and management strategies are not implemented [4].

The etiology of AD remains incompletely understood, with the condition widely recognized as a complex, multifactorial disorder influenced by aging, environmental exposures, and genetic predisposition [5,6,7]. Traditionally, two primary and partially complementary hypotheses have been proposed to explain its pathogenesis. The first is the amyloid cascade hypothesis [8], which posits that the aberrant accumulation of extracellular amyloid-beta (Aβ) peptides represents a central pathological hallmark of AD. These peptides are produced through the sequential proteolytic cleavage of amyloid precursor protein (APP) by β- and γ-secretases [9]. The second major framework is the tau hypothesis. Although not exclusive to AD, as similar tau pathologies are present in disorders such as frontotemporal dementia, progressive supranuclear palsy, corticobasal degeneration, and Pick’s disease, it proposes that tau pathology initiates in specific brain regions, such as the entorhinal cortex or locus coeruleus (LC), and subsequently propagates through neuronal circuits to involve widespread cortical areas, ultimately resulting in neurodegeneration [10,11].

Current understanding suggests that AD progression is marked by a gradual topographic decline in the function of multiple neurotransmitter systems, including cholinergic (acetylcholinergic), catecholaminergic (dopaminergic, noradrenergic), and indoleaminergic (serotonergic) pathways, accompanied by extensive neuronal loss [12]. This neurodegeneration is preceded by two pathological hallmarks: the extracellular deposition of neurotoxic Aβ, forming neuritic plaques in the neocortex, and the intracellular aggregation of hyperphosphorylated tau protein, resulting in neurofibrillary tangle formation [13,14]. In addition to these hallmark features, several other pathophysiological mechanisms have been implicated in AD, including cerebral hypoperfusion, chronic neuroinflammation, disrupted calcium homeostasis, and mitochondrial dysfunction [14,15,16]. A growing body of evidence also points to the involvement of additional contributing factors, such as aberrant glycosylation of lipids and proteins, blood–brain barrier (BBB) breakdown, impaired mitochondrial bioenergetics, oxidative stress, and sustained neuroinflammatory responses [17,18,19]. Moreover, biomedical research increasingly highlights the potential influence of the gut microbiome (GM) in host cognitive function and in a range of pathological processes, including anxiety, depression, schizophrenia, and stress-related disorders [20,21,22]. In this respect, emerging evidence suggests that GM dysbiosis, which is characterized by shifts in microbial composition, reduced diversity, and altered functionality, may modulate neural circuits involved in cognitive decline and neurodegeneration [23,24], making it a key factor in AD pathology [25,26].

Although accumulating evidence underscores the potential role of the GM in the pathogenesis of AD, the exact contribution of GM dysbiosis remains ambiguous. It is still unclear whether dysbiosis acts as a causal factor driving AD pathology or emerges as a consequence of disease-associated changes. A growing body of research highlights the fecal metabolome as a functional proxy for GM activity [27], suggesting that microbial metabolites may constitute a molecular interface linking the GM to brain function [28]. Metabolomics, which involves the high-throughput profiling and quantification of small molecules within biological specimens, provides a powerful approach for identifying novel disease biomarkers [29,30]. Recent metabolomics investigations have identified several metabolic pathways implicated in AD pathophysiology [31,32]. Furthermore, there is a discernible shift in GM-focused investigations, moving from primarily association-based studies toward the exploration of therapeutic interventions. In this respect, decades of observational research have underscored the potential of GM modulation as a promising therapeutic avenue for addressing AD [33]. Accordingly, investigating the microbiota–gut–brain (MGB) axis is crucial for identifying novel therapeutic targets and developing innovative treatment strategies for AD.

Building on the aforementioned, the relationship between GM dynamics and AD constitutes an area of increasing scientific interest, although much remains to be defined. Clarifying and highlighting the functional relevance of microbial activity, particularly at the molecular level, may help frame new perspectives on disease mechanisms and interventions, which is essential given the complex nature of AD and the urgent need for innovative therapeutic strategies. This narrative review examines current evidence linking GM dysbiosis to AD, focusing on microbial metabolomes and their mechanistic role, as well as on the potential of GM-targeted therapeutic approaches.

## 2. Method

A narrative, non-systematic examination of the literature was conducted to provide a comprehensive overview of the subject under examination. Database searches were performed in PubMed, Scopus, and Web of Science between June and July 2025. The search strategy employed an iterative process combining various relevant keywords related to AD, the GM, microbial metabolites, GM-host interactions, and GM-targeted therapeutics. Specifically, the search strings were applied to the title and abstract fields, including terms such as “Alzheimer’s disease”, “gut microbiome”, “microbial metabolites”, “gut–brain axis”, “probiotics”, “prebiotics”, and “fecal microbiota transplantation”. No restrictions or filters were applied regarding publication date, study type, population, or language. However, only articles published in English and Spanish were considered, without the need for translation tools. In addition, articles known for their relevance were included, and reference lists from pertinent studies were screened to identify further significant sources. The selection process involved two stages. First, titles and abstracts were screened to exclude studies not aligned with the central research focus. Second, full texts of the remaining articles were examined in detail. Inclusion criteria prioritized studies addressing GM dysbiosis, the role of microbial metabolites, and therapeutic approaches targeting the GM, all specifically within the context of AD. Exclusion criteria included studies without accessible full texts, conference abstracts, letters, theses, and research not directly or indirectly addressing the GM-AD relationship. Extracted data were then thematically synthesized to structure the review, thereby providing a cohesive compilation of current knowledge pertinent to these domains.

## 3. Alzheimer’s Disease and Gut Microbiome Dysbiosis

### 3.1. Alteration in Gut Microbiome Composition in Alzheimer’s Disease

Acknowledging the connection between the GM and AD, recent studies have focused on characterizing this relationship [25,34]. The GM profiles of individuals with AD differ markedly from those of healthy controls, particularly in terms of microbial diversity and the relative abundance of specific bacterial taxa, which vary according to geographic region and the clinical stage of the disease. The current literature consistently reports a reduction in bacterial diversity in AD, as well as notable alterations at the phylum, family, and genus levels [32,35,36,37,38,39,40,41,42,43,44,45]. While the majority of studies report converging findings, discrepancies have been observed in certain bacterial families, such as *Bacteroidaceae*, *Rikenellaceae*, and *Ruminococcaceae*, which yielded conflicting results across some investigations [38,43,45]. Similar inconsistencies are noted at the genus level for *Alistipes*, *Alloprevotella*, *Barnesiella*, *Bifidobacterium*, *Blautia*, *Coprococcus*, *Eubacterium*, *Faecalibacterium*, and *Ruminococcus* [32,38,39,41,43]. Table 1 presents significant differences in the prevalence of GM bacterial taxa between AD patients and cognitively healthy individuals.

Despite considerable inter- and intra-individual variability influenced by factors such as sex, diet, and geographic region, studies have shown that approximately 85% of AD patients exhibit a distinct GM profile compared to age-matched healthy controls [32,46]. It has been stated that GM dysbiosis observed in AD patients may critically impact the MGB axis and contribute actively to AD pathogenesis [47]. Several clinical investigations have explored associations between specific gut microbial taxa and AD biomarkers or disease progression. For instance, Cattaneo et al. [35] reported an increased abundance of the pro-inflammatory genera *Escherichia/Shigella* and a decreased abundance of the anti-inflammatory species *Eubacterium rectale*, findings that correlated with elevated peripheral inflammation markers. They found significant positive correlations between *Escherichia/Shigella* abundance and pro-inflammatory cytokines IL-1β, NLRP3, and CXCL2, whereas *E. rectale* exhibited negative correlations with these cytokines. Similarly, Vogt et al. [43] documented reduced microbial diversity and altered GM composition in AD patients compared with controls, including decreases in the phyla Actinomycetota and Bacillota, as well as lower abundances of families such as *Clostridiaceae*, *Mogibacteriaceae*, *Peptostreptococcaceae*, *Ruminococcaceae*, and *Turicibacteraceae*, and genera including *Adlercreutzia*, *Bifidobacterium*, *Bilophila*, *Clostridium*, *Dialister*, and *Turicibacter*. Conversely, they observed increased abundance of taxa within Bacillota phylum and in families and genera such as *Bacteroidaceae*, *Gemellaceae*, *Rikenellaceae*, *Alistipes*, *Bacteroides*, *Blautia*, *Gemella*, and *Phascolarctobacterium*. Zhuang et al. [45] reported altered fecal microbiota composition in AD characterized by decreased Actinomycetota and increased Bacteroidota phyla. Increases were also noted in families *Enterococcaceae*, *Lactobacillaceae*, and *Ruminococcaceae*, as well as in genera *Bacteroides*, *Ruminococcus*, and *Subdoligranulum*, while the abundance of *Bacteroidaceae*, *Lachnoclostridium*, *Lachnospiraceae*, and *Veillonellaceae* decreased. Ling et al. [40] observed a marked reduction in bacterial diversity in Chinese AD patients, with decreased abundance of butyrate-producing genera such as *Butyricicoccus*, *Coprococcus*, *Faecalibacterium*, *Gemmiger*, and *Roseburia*, alongside increased levels of the propionate-producing *Akkermansia* and of the lactate-producing *Bifidobacterium*. In addition, genera including *Clostridium*, *Dialister*, and *Romboutsia* were diminished in AD. Hung et al. [38], in a systematic review encompassing 11 studies, reported that patients with AD, but not those with mild cognitive impairment (MCI), exhibited significantly lower GM diversity compared with healthy controls, and noted geographical variation in microbial profiles. The Pseudomonadota phylum and genera *Bifidobacterium* and *Phascolarctobacterium* were relatively predominant, whereas Bacillota phylum members and families such as *Clostridiaceae*, *Lachnospiraceae*, and *Rikenellaceae* were relatively less abundant. More recently, Heravi et al. [37] highlighted altered GM signatures in AD patients compared with healthy controls, characterized by elevated proportions of phyla Acidobacteriota and Actinomycetota, as well as family *Ruminococcaceae* and genus *Bacteroides*. Conversely, reductions were observed in Bacillota phylum, families *Acidaminococcaceae* and *Lachnospiraceae*, and genus *Ruminiclostridium*.

Nevertheless, some studies have reported conflicting findings, showing no significant alterations in the GM of AD patients compared to controls. For instance, Cirstea et al. [48] found that although the GM diversity was reduced in AD patients, the overall microbial composition did not differ significantly from that of the control group. Furthermore, elevated levels of the phylum Bacteroidota have been observed both in cognitively normal controls with Aβ-positive plasma [49], and in AD patients [36,43,45]. Additionally, as highlighted by Jemimah et al. [50], the abundance of genera *Bacteroides* and *Phascolarctobacterium* in AD patients appears to be modulated by geographic factors, with diet and lifestyle playing a pivotal role in these variations.

### 3.2. Relationship Between the Gut Microbiome and Biomarkers in Alzheimer’s Disease

Several clinical studies have investigated the association between specific gut microbial taxa and clinical biomarkers of AD progression. For instance, Cattaneo et al. [35] identified a positive correlation between increased levels of *Escherichia/Shigella* and markers of peripheral inflammation as well as brain amyloidosis. Vogt et al. [43] reported associations between certain gut genera and cerebrospinal fluid (CSF) biomarkers of AD, including Aβ42/Aβ40 ratios, chitinase-3-like protein 1, phosphorylated tau (p-tau), and the p-tau/Aβ ratio. Specifically, they found that elevated levels of *Bacteroides* and *Blautia* correlated positively with p-tau and the p-tau/Aβ42 ratio. On the other hand, Sheng et al. [49] observed a negative correlation between plasma Aβ42 and Aβ42/Aβ40 ratios and the abundance of the family *Desulfovibrionaceae* and the genera *Bilophila* and *Faecalibacterium*, suggesting that higher levels of these taxa may be linked to reduced amyloid deposition in the brain. In addition, postmortem analyses of AD brain tissue have revealed co-localization of lipopolysaccharides (LPS) and *Escherichia coli* with amyloid plaques, implying a potential mechanistic link between GM components and amyloid pathogenesis [39]. This evidence supports the hypothesis that amyloid pathology in AD may be initiated during MCI as a consequence of shifts in the GM. Nevertheless, the causal involvement of these bacterial components in AD development, as well as the extent of their presence, remains a matter of debate within the scientific community. Several factors contribute to this controversy: (i) a causal relationship has not been definitively established; (ii) LPS-induced animal models of AD have inherent limitations, as they may not fully capture the complex pathophysiological mechanisms of human neurodegenerative diseases; and (iii) the co-localization of bacterial components with plaques demonstrates a correlation, but does not provide conclusive evidence of direct causation. Additional hypothesis might be considered. In this context, antibodies may be used to detect Aβ deposits in AD. However, antibody specificity is crucial for accurately identifying plaques and distinguishing them from artifacts. Misfolded proteins, for instance, may be erroneously interpreted as plaques, while low specificity can result in false positives or negatives. Consequently, the use of highly specific antibodies targeting modified or deposited forms of Aβ, rather than general Aβ antibodies, is essential for precise diagnosis and for elucidating the role of Aβ in AD pathogenesis.

Additional research has focused on delineating differences in GM profiles between MCI and AD, in order to clarify their relationship with disease progression. In a U.S. longitudinal cohort study of 108 nursing home elders (ages 65–94) with a 5-month follow-up, where shotgun metagenomics (NextSeq 500) was used, Haran et al. [36] found that GM composition varies by dementia subtype. AD patients showed increased abundance of *Alistipes*, *Bacteroides*, *Barnesiella*, and *Odoribacter*, as well as decreased abundance of *Lachnoclostridium*. Other dementia types were characterized by increased *Barnesiella* and *Odoribacter*, but decreased *Collinsella*, *Eubacterium*, *Lachnoclostridium*, and *Roseburia*. In China, a prospective cross-sectional study including 97 subjects (33 with AD, 32 with amnestic MCI, and 32 healthy controls, aged 50–85 years), where 16S rRNA sequencing was used, Liu et al. [41] reported a reduction in fecal microbial diversity in AD patients compared to both MCI and control groups, with significant compositional differences. The families *Clostridiaceae* and *Lachnospiraceae* were markedly decreased in both AD and MCI groups relative to controls, while *Blautia*, *Ruminococcus*, and the family *Ruminococcaceae* showed further decline in AD versus MCI. Moreover, a progressive increase in *Enterobacteriaceae* abundance was noted from controls to AD patients, whereas *Veillonellaceae* abundance increased from MCI to controls. Li et al. [39], in a cross-sectional study conducted in China including 90 subjects (30 with AD, 30 with MCI, and 30 healthy controls, with a mean age of 65.5 years), using 16S rRNA gene sequencing, also demonstrated that microbial diversity was diminished in both AD and MCI patients, with no significant differences between these two groups. However, they identified 11 genera that differed between AD patients and healthy controls across fecal and blood samples. Genera such as *Bifidobacterium*, *Blautia*, *Dorea*, *Escherichia*, *Lactobacillus*, and *Streptococcus* were increased in AD, while genera such as *Alistipes*, *Bacteroides*, *Parabacteroides*, *Paraprevotella*, and *Sutterella* were decreased.

Complementary studies have explored the relationship between variations in GM profiles and factors such as disease severity [51,52], the presence of neuropsychiatric symptoms [53], and an increased likelihood of positive amyloid and p-tau status [54]. For instance, Guo et al. [51] performed a comparative analysis of fecal samples from individuals with AD, MCI, and healthy controls. Their results showed no significant differences in microbial α-diversity among the groups, but β-diversity was elevated in both AD and MCI patients. Compared to healthy controls, AD patients exhibited decreased abundances of *Bacteroides*, *Lachnospira*, and *Ruminiclostridium*, as well as an increase in *Prevotella*. MCI patients displayed a similar pattern of alteration when compared to AD patients. However, *Lachnospira* was the only genus significantly reduced in MCI compared to controls. Notably, the negative correlation between *Prevotella* abundance and cognitive function persisted in the MCI group. These findings suggest that while the overall richness and evenness of microbial communities (α-diversity) may remain relatively stable in early to moderate stages of cognitive decline, β-diversity often differs because it reflects changes in community composition and structure associated with AD-related neurodegeneration and metabolic alterations.

Yıldırım et al. [52] reported that individuals with AD or MCI exhibit GM dysbiosis characterized by a reduction in beneficial bacteria such as *Bacteroides*, in addition to an increase in pro-inflammatory genera including *Prevotella*. The genera *Faecalibacterium*, *Fusicatenibacter*, *Lactobacillus*, and *Roseburia* were found to be decreased in abundance, whereas *Akkermansia* and *Escherichia/Shigella* were more prevalent in AD samples. Verhaar et al. [54] conducted a comparative analysis of GM compositions among patients with AD, MCI, and subjective cognitive decline, identifying only two genera, *Phascolarctobacterium* and *Subdoligranulum*, with significantly different abundances across groups, while α- and β-diversity metrics showed no notable differences. Their findings highlighted that the most prominent predictors of amyloid and p-tau status were members of the *Lachnospiraceae* family, including *Lachnoclostridium*, *Marvinbryantia*, *Monoglobus*, *Roseburia*, and *Ruminococcus*. In contrast, elevated levels of *Alistipes* and *Odoribacter* correlated with biomarker profiles indicative of more typical AD pathology, such as increased amyloid and decreased p-tau in CSF. Furthermore, key predictors of amyloid levels included *Anaerostipes*, *Eubacterium*, and *Subdoligranulum*, while p-tau predictors encompassed *Lachnospiraceae* family members *Blautia* and *Lachnoclostridium*. Wanapaisan et al. [55] observed no significant differences in α- and β-diversity between AD, MCI, and control groups in a Thai cohort. However, bacterial abundance varied, as *Lachnospiraceae* family members and *Clostridium* genus were decreased in both AD and MCI compared to controls. In addition, AD patients showed increased levels of *Bacteroides*, *Escherichia/Shigella*, *Holdemanella*, *Megamonas*, and *Romboutsia*, whereas MCI patients exhibited higher abundances of *Agathobacter*, *Faecalibacterium*, and *Fusicatenibacter*. The authors suggested that reductions in *Agathobacter*, *Clostridium*, and *Faecalibacterium* in patients with AD may correlate positively with amygdala and hippocampal brain volumes, regions known to undergo atrophy during early cognitive decline [56].

### 3.3. The Role of the Gut Microbiome in the Pathogenesis of Alzheimer’s Disease

As previously stated, two primary hypotheses have been traditionally proposed to elucidate the etiology and pathogenic mechanisms of AD. The amyloid cascade hypothesis [8,57] suggests that the accumulation of Aβ plaques within the nervous system constitutes a central risk factor for AD. Specifically, the aggregation of neurotoxic Aβ42 peptides in the brain is recognized as a critical event leading to amyloid plaque formation [58]. This Aβ aggregation initiates a neurotoxic cascade, which triggers cytoskeletal disruptions, neuronal dysfunction, and ultimately neuronal death [59]. Studies have identified the Toll-like receptor 2 (TLR2)-myeloid differentiation primary response 88 (MyD88) signaling pathway as playing a pivotal role in Aβ generation [60]. Experimental evidence in murine models shows that deficiency of MyD88 enhances Aβ clearance via low-density lipoprotein receptor-related protein 1 (LRP1) [61], thereby reducing pro-inflammatory mediators and brain Aβ accumulation [62]. This reduction has been correlated with improved cognitive performance in rat models. Nevertheless, some studies have questioned the essentiality of MyD88 signaling, suggesting that MyD88-dependent pathways may not be strictly required for neuroglial activation or the development of Aβ pathology in the brain [63].

Another crucial hypothesis concerning AD pathogenesis centers on the hyperphosphorylation of tau protein, which leads to the formation of neurofibrillary tangles [64]. Tau protein plays an important role in promoting microtubule assembly and maintaining their stability [65]. Its hyperphosphorylation disrupts synaptic plasticity, which is essential for coordinating memory-related pathways [66]. Thus, tau hyperphosphorylation is directly implicated in triggering the neurodegenerative processes characteristic of AD [67]. Pathogenic tau also induces the release of mitochondrial DNA from microglia, which activates the antiviral cGAS–IFN signaling pathway, resulting in sustained interferon release. This persistent immune activation impairs synaptic integrity and plasticity, ultimately contributing to ongoing cognitive deficits [68]. Recent evidence highlights phosphorylated tau protein 217 as a promising biomarker for AD diagnosis and progression monitoring [69]. Building on these insights, the “dual prion disorder” hypothesis has been proposed, which posits that the pathological interplay between Aβ and tau proteins represents a viable therapeutic target [70,71]. In addition to the well-established amyloid cascade and tau phosphorylation hypotheses, other significant theories explore the roles of neuroinflammation [72], mitochondrial dysfunction [73], and cholinergic deficits [74] in AD pathogenesis. Collectively, these diverse perspectives reflect the ongoing efforts within the scientific community to reveal the complex mechanisms underlying AD.

A growing body of human research has identified several bacterial species, including *Borrelia burgdorferi*, *Chlamydia pneumonia*, and *Helicobacter pylori*, as factors that may increase susceptibility to AD by promoting tau protein hyperphosphorylation, elevating pro-inflammatory bacteria such as *Escherichia/Shigella*, and reducing anti-inflammatory gut microorganisms such as *Ruminococcus* [75,76]. Bostanciklioğlu [15] proposed three primary mechanisms linking the GM to AD pathogenesis: (i) central nervous system (CNS) inflammation and cerebrovascular degeneration triggered by bacterial metabolites and amyloids; (ii) impaired autophagy-mediated protein clearance caused by GM dysbiosis; and (iii) modulation of brain neurotransmitter levels via vagal afferents influenced by the GM. Accumulating evidence indicates that disturbances in human GM homeostasis may contribute to the deposition of tau and Aβ peptides [43,77,78]. Multiple gut microbial species, including *Bacillus subtilis*, *E. coli*, *Salmonella enterica*, and *S. enterica* serotype Typhimurium, have been shown to produce amyloid fibers [79,80,81,82,83]. While microbial amyloids share only tertiary structural similarities with human CNS amyloids, they act as prion-like agents through molecular mimicry, inducing pathogenic β-sheet conformations in host amyloidogenic proteins [16,84,85,86,87]. In elderly individuals, potential mechanisms of amyloid propagation include neuron-to-neuron or distal neuronal spread, as well as direct crossing of the BBB or transport via other cells, such as astrocytes, fibroblasts, microglia, and immune cells [16]. Furthermore, Aβ, tau, and α-synuclein may contribute to neurodegeneration by inducing or exacerbating BBB disruption. These pathological proteins can compromise BBB integrity either directly, by affecting key components such as pericytes and endothelial cells, or indirectly via the activation and dysfunction of brain macrophages [88].

Systemic inflammatory responses triggered by bacterial secretions or structural components have been implicated in the disruption and increased permeability of the BBB. This process involves activation of the TLR4/IRF-3 signaling pathway within endothelial cells lining cerebral blood vessels. Similarly, systemic inflammation induced by bacterial products has been linked to impairment of the intestinal epithelial barrier integrity [89]. Moreover, activation of the lipopolysaccharide (LPS)/TLR4 pathway in glial cells within the CNS has been shown to promote neuroinflammation [90]. Bacterial LPS presence in the brain, facilitated by translocation across both the intestinal barrier and the BBB, has been implicated in AD pathogenesis [91]. LPS stimulates leukocyte and microglial receptors, including TLR4-CD14 and TLR2, resulting in elevated cytokine production and increased Aβ accumulation. In addition, Aβ1–42 acts as an agonist for TLR4 receptors, activating NF-κB pathways that further amplify cytokine release, exacerbating Aβ accumulation, damaging oligodendrocytes, and contributing to the myelin degeneration observed in AD brains [92]. In a recent hypothesis proposed by Brown and Heneka [93], LPS is suggested to contribute to AD pathophysiology through peripheral infections or GM dysbiosis, which elevate LPS concentrations in circulation and the brain, thereby promoting amyloid and tau pathologies along with microglial activation and subsequent neurodegeneration characteristic of AD. Supporting evidence for this hypothesis includes: (i) elevated LPS levels detected in the blood and brains of AD patients; (ii) AD risk factors associated with increased LPS levels or heightened responses; (iii) LPS-induced upregulation, aggregation, inflammation, and neurotoxicity of Aβ; (iv) LPS-driven tau phosphorylation, aggregation, and propagation; (v) LPS-mediated microglial priming, activation, and neurotoxicity; and (vi) LPS-induced synaptic and neuronal loss and memory impairment in AD mouse models, as well as cognitive deficits observed in humans.

The MGB axis plays a pivotal role in the pathophysiology of AD, with its underlying mechanisms extensively characterized [94]. Alterations in GM diversity and their metabolic products in AD disrupt the homeostatic balance of the GM, leading to impaired communication between microorganisms, the gut epithelium, and the immune system, ultimately resulting in developmental and functional deficits of the gut-immune barrier [95]. Metabolites derived from the GM exacerbate damage to the gut mucosal immune system, increase intestinal permeability (commonly referred to as “leaky gut”), and promote abnormal bacterial translocation [96]. These changes intensify systemic inflammatory responses, characterized by a dysregulated Th17/Treg cell ratio and elevated circulating levels of pro-inflammatory cytokines including IL-6, IL-1β, IFN-γ, and TNF-α. Collectively, these immune alterations contribute significantly to the pathological processes associated with AD [95,97,98].

## 4. Microbial Metabolomes

The bacterial metabolome generated by the GM can influence brain function through several mechanisms: (i) the production of neurotransmitters and neuromodulators such as acetylcholine (ACh), γ-aminobutyric acid (GABA), dopamine, histamine, melatonin, norepinephrine, and serotonin, a process further enhanced by activated catecholamines within the gut lumen [99]; (ii) the synthesis of bioactive metabolites, including bile acids (BAs) and short-chain fatty acids (SCFAs), which interact with enteroendocrine and enterochromaffin cells, the mucosal immune system, and the intestinal barrier, allowing these metabolites to enter systemic circulation, with some crossing the BBB via organic anion transporters in the case of BAs and monocarboxylate transporters for SCFAs [46]; (iii) the regulation of tryptophan (Trp) metabolism, impacting levels of serotonin, kynurenic acid (KYN), and quinolinic acid [100]; and (iv) the production of pro-inflammatory and anti-inflammatory cytokines, which may indirectly activate the hypothalamic–pituitary–adrenal (HPA) axis to stimulate the release of adrenocorticotropin hormone, corticotropin-releasing hormone, and cortisol [101], or alternatively, these cytokines may exert direct effects on CNS immune activity [75]. Table 2 shows the role of microbial compounds involved in the pathogenesis of AD.

**Table 2:** Role of microbial compounds involved in the pathogenesis of AD.

Microbial Compounds	Bacterial Producers	Major Effects	References
Sphingolipids	*Bacteroides*, members of Pseudomonadota.	- Restore the intestinal mucosal barrier. - Regulate microglia. - Induce neuroinflammation	[102,103,104]
Phospholipids	*Akkermansia*, *Desulfovibrio*.	- Associated with immune system activation, mitochondrial function, neuroinflammation, and oxidative stress.	[105,106,107]
LPS	*Bacteroides*, *Escherichia/Shigella*.	- Microglial activation. - Release pro-inflammatory factors. - Regulate Aβ accumulation.	[93,108]
SCFAs	*Akkermansia*, *Bacteroides*, members of Bacillota.	- Inhibit tau protein phosphorylation. - Reduce oxidative stress. - Release pro-inflammatory factors. - Regulate mitochondrial homeostasis.	[109,110,111]
BAs	*Bacteroides*, *Bifidobacterium*.	- Regulate tau and Aβ accumulation. - Promote mitochondrial biogenesis.	[112,113,114,115]
Amino acids	*Clostridium*, *Escherichia*, *Lactobacillus*.	- Activate oxidative stress. - Regulate microglial polarization. - Reduce oxidative stress.	[116,117,118]
GABA	*Bacteroides*, *Bifidobacterium*, *Escherichia*, *Lacticigenium*.	- Promotes the spread of tau and Aβ pathologies. - Promotes neuronal differentiation.	[119,120,121]
Serotonin	*Bifidobacterium*, *Lacticigenium*, *Roseburia*.	- Increases vagus neuron activity. - Regulates astrocyte and microglia activities. - Reduces Aβ aggregation and tau phosphorylation.	[122,123,124]
ACh	*Bacillus*, *Escherichia*, *Lactiplantibacillus*, *Staphylococcus*.	- Promotes the deposition of Aβ plaques. - Induces hippocampal atrophy.	[125,126]
Dopamine	*Bacillus*, *Bacteroides*, *Bifidobacterium*, *Brevilactibacter*.	- Regulates the intensity of synapses in neurons. - Affects cognition and mood.	[127,128,129]
Norepinephrine	*Bacillus*, *Escherichia*, *Proteus*.	- Regulates synaptic plasticity. - Upregulates BDNF. - Reduces pro-inflammatory factors. - Increases amyloid clearance.	[130,131,132]

LPS: lipopolysaccharides; Aβ: amyloid-beta; SCFAs: short-chain fatty acids; BAs: bile acids; GABA: gamma-aminobutyric acid; ACh: acetylcholine; BDNF: brain-derived neurotrophic factor.

The GM can influence the CNS by producing specific metabolites that are transported via the adrenal glands or the vagus nerve and cross the BBB, thereby impacting brain cells either directly by modulating neuronal behavior or indirectly by inducing epigenetic modifications in chromatin [101,133]. Numerous functional changes triggered by microbial metabolites have been linked to cognitive impairments [134,135]. In patients with AD, increased folate biosynthesis alongside reduced fatty acid and butyrate production has been reported [24,25]. In addition, specific gut metabolomic signatures, including BAs, SCFAs, and Trp metabolites, have shown correlations with the severity of cognitive decline [47]. Distinct differences in fecal metabolites, such as benzenoids, lipids, organic acids, and piperidine, have been proposed as potential biomarkers distinguishing AD patients from healthy individuals [32,136]. Importantly, dysbiotic GM byproducts can alter gene expression and the levels of synaptic proteins, which may promote accumulation of inflammatory proteins in the brain, trigger neuroinflammation, activate astrocytes, induce neuronal apoptosis, and provoke microglial inflammatory responses [137,138]. Furthermore, several investigations have documented increased lipid peroxidation alongside decreased concentrations and activity of antioxidant molecules in AD patients [139,140].

### 4.1. Bacterial Components

Sphingolipids, classified as bioactive lipids, are essential to cellular signal transduction pathways [141]. These molecules regulate numerous cellular functions, including neuronal stress responses, cell proliferation, differentiation, and maturation [142]. Host sphingolipids promote the release of pro-inflammatory mediators that impact neuronal viability and mitochondrial apoptosis, thereby contributing to neuroinflammation processes implicated in various brain disorders [104]. Emerging evidence suggests that bacterial-derived sphingolipids can stimulate the gut immune system and metabolic homeostasis. However, evidence regarding their direct impact on the human CNS remains limited. Metabolomic profiling of brain tissue and blood samples during preclinical and prodromal phases of AD reveals mixed patterns, with some ceramides increased and certain sphingomyelin levels decreased, which is associated with the progression of AD pathology [143,144]. Moreover, sphingolipid concentrations have been correlated with CSF Aβ levels, brain atrophy, and cognitive deterioration, underscoring their potential utility as early biomarkers for AD [143].

Phospholipids form the fundamental components of the cellular membrane lipid bilayer, serving as a crucial protective barrier for both cellular and subcellular structures. Beyond this structural role, they are involved in maintaining cellular homeostasis, regulating immune responses, and modulating oxidative stress and neuroinflammation within the brain [28]. The predominant phospholipids in the brain are phosphatidylcholine and phosphatidylethanolamine, which relative abundances are altered in the context of AD pathology [145]. The catabolism of these phospholipids is considered a significant metabolic disturbance in AD, with decreased levels strongly correlated with the extent of amyloid deposition and neurofibrillary tangle formation [146].

A recent investigation identified the presence of LPS, which constitute endotoxins produced by Gram-negative bacteria such as *Bacteroides fragilis*, *E. coli*, and *S. enterica*, within amyloid plaques in postmortem brain tissues from individuals with AD [147]. This discovery suggests a possible co-occurrence of these LPS-producing bacteria alongside *E. coli* in the pathogenesis of AD. In addition, elevated LPS levels have been detected in the blood of AD patients, as well as in the neocortex and hippocampus, where concentrations exceed those found in control subjects [148]. These findings implicate LPS as a potential risk factor contributing to cognitive decline and AD progression [149]. LPS has been shown to compromise both the intestinal barrier and the BBB, triggering a potent neuroinflammatory response, promoting Aβ deposition, and inducing abnormal tau hyperphosphorylation [93]. Such effects lead to microglial activation and neuroinflammation [150], as well as oxidative stress and mitochondrial dysfunction mediated by NADPH oxidase 2 (NOX2), culminating in neuronal injury and synaptic degeneration [149]. Furthermore, LPS acts as an agonist for Toll-like receptor 4 (TLR4), a critical receptor in microglial innate immunity [151], thereby facilitating the release of pro-inflammatory cytokines and further enhancing Aβ accumulation [28].

### 4.2. Bioactive Microbial Metabolites

#### 4.2.1. Short-Chain Fatty Acids

SCFAs are key metabolites produced by the GM that play essential roles in maintaining human physiological homeostasis, including anti-inflammatory effects, energy provision, gut barrier integrity, and immune regulation [109]. SCFAs are generated via enzymatic degradation of carbohydrates and plant polysaccharides by the GM, with acetate, butyrate, propionate, and valerate as the primary SCFAs. Overall, members of the phyla Bacillota and Bacteroidota mainly produce propionate, while specific genera such as *Anaerostipes*, *Eubacterium*, *Faecalibacterium*, and *Roseburia*, all belonging to the Bacillota phylum, are predominant butyrate producers. The bacterium *Akkermansia muciniphila* uniquely utilizes intestinal mucin as its carbon and nitrogen source, generating propionate as a key metabolite, and *Megasphaera* species primarily produce valerate [152].

These SCFAs can cross the BBB or signal via the MGB axis, influencing gene expression, lipid metabolism, mitochondrial function, and neurotransmitter synthesis. SCFAs could modulate relevant processes in AD by acting as ligands for specific G protein-coupled receptors (GPCRs), thereby affecting intracellular signaling pathways [153]. Identified SCFA receptors include GPR41 (FFAR3), GPR42, GPR43 (FFAR2), GPR109A (HCAR2), GPR164 (OR51E1), and OR51E2 (Olfr78) [154]. Of these, SCFAs particularly bind to GPR43 and GPR109A, leading to regulation of cholesterol and lipid metabolism, activation of the MAPK signaling pathway, and suppression of the pro-inflammatory NF-κB pathway [111]. This receptor-mediated signaling highlights the pivotal role of SCFAs in modulating neuroinflammation and metabolic dysfunctions associated with AD.

A growing body of research has established a correlation between specific SCFAs, such as acetate and valerate, and amyloid deposition in the brain, alongside elevated pro-inflammatory cytokines in individuals with AD [155]. Butyrate, in particular, influences serotonin release within the enteric nervous system, which can stimulate the vagus nerve and activate endocrine signaling pathways that affect brain function [156]. Epigenetic modulation represents a key mechanism by which SCFAs impact AD pathology. Butyrate acts as a histone deacetylase (HDAC) inhibitor, increasing the expression of genes linked to learning, restoring histone acetylation, and significantly improving learning and memory abilities in neurodegenerative conditions [157]. Similarly, acetate enhances cognition by promoting histone H3K18 acetylation [158].

SCFAs have also been shown to suppress the secretion of pro-inflammatory cytokines, including IL-1β, MCP-1, and TNF-α, and reduce the phagocytic activity of THP-1 cells [159]. Their anti-inflammatory properties involve several pathways. (i) acetate supplementation reverses LPS-induced upregulation of phospholipase C β1, COX-1, and COX-2 [160]; (ii) butyrate inhibits COX-2 expression in Aβ-treated BV2 microglial cells, accompanied by decreased NF-κB p65 phosphorylation [161]; and (iii) the AKT-Rho GTPase signaling pathway mediates the effect of sodium butyrate on microglial process elongation [162]. These regulatory effects are dependent on HDAC inhibition, which promotes the binding of acetylated histone H3K9 to promoter regions of target genes, facilitating transcription [162]. Furthermore, SCFAs modulate astrocyte-mediated inflammation through multiple mechanisms. Acetate supplementation reduces pro-inflammatory cytokines TNF-α and IL-6 by downregulating p38 MAPK and NF-κB signaling, while increasing anti-inflammatory IL-4 levels by upregulating TGF-β1 signaling. These effects may be linked to enhanced acetylation of H3K9 [163].

**Figure 1:**
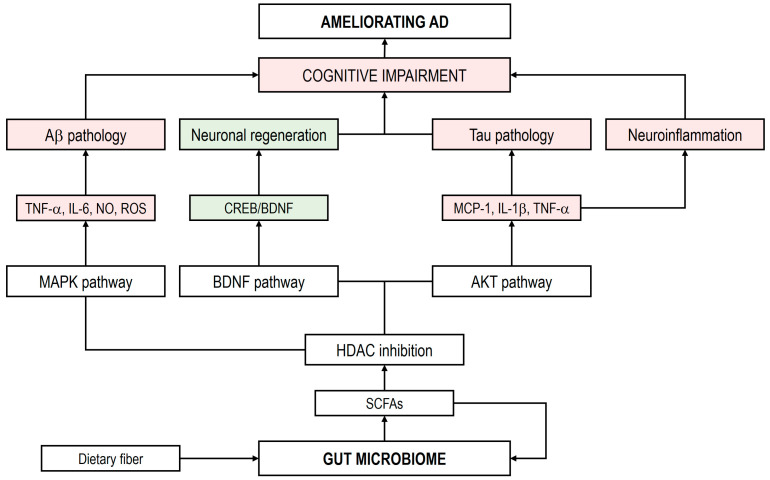
Hypothetical overview of the effects of SCFAs in AD. SCFAs produced by the GM cross the BBB to enter the CNS. Within the CNS, SCFAs act on neurons to promote regeneration by upregulating the CREB/BDNF signaling pathway and enhancing the expression of genes involved in memory consolidation. Simultaneously, SCFAs attenuate the release of pro-inflammatory mediators by inhibiting signaling pathways such as MAPK and NF-κB in activated microglia and astrocytes. Moreover, SCFAs modulate pathological alterations in Aβ and tau proteins, ultimately contributing to the improvement of cognitive function in AD. Rectangles in green: increase. Rectangles in red: decrease. Abbreviations: HDACs, histone deacetylases; AKT, protein kinase B; CREB, cyclic-AMP response element binding protein; BDNF, brain-derived neurotrophic factor; NF-κB, nuclear factor-κB; MAPK, mitogen-activated protein kinase; NO, nitric oxide; ROS, reactive oxygen species.

Preclinical studies have demonstrated that butyrate positively influences synaptic plasticity by increasing synapse-associated proteins and facilitating long-term potentiation and depotentiation [164]. In microglia, sodium butyrate upregulates the PI3K/AKT/CREB/BDNF signaling pathway, which supports synaptic plasticity and long-term potentiation [165]. In addition, SCFAs have been shown to promote the mitosis of human neural progenitor cells by modulating gene expression related to proliferation, apoptosis, and neurogenesis, thereby contributing to neural regeneration [166]. Figure 1 provides a hypothetical overview of the effects of SCFAs in AD (adapted from Huang et al. [110] and Qian et al. [111]).

#### 4.2.2. Bile Acids

BAs can be subdivided into primary and secondary BAs. Primary BAs include chenodeoxycholic acid and cholic acid [134]. In the intestinal tract, bile salt hydrolases produced by bacterial genera such as *Bacteroides*, *Bifidobacterium*, *Clostridium* cluster VIa, and *Lactobacillus* convert primary BAs into secondary BAs, including deoxycholic acid, lithocholic acid, and ursodeoxycholic acid [167]. Primary BAs play essential roles in lipid absorption and cholesterol homeostasis and can cross the BBB to bind nuclear receptors, thereby modulating brain physiological functions. Alterations in BA profiles have been associated with AD and cognitive decline, characterized by elevated levels of the secondary BA deoxycholic acid and reduced levels of primary BAs such as cholic acid, suggesting a link between BA metabolism and cognitive function [167,168]. Increased secondary BA levels have also been implicated in Aβ production and accumulation via disruption of cholesterol catabolism [169].

A recent fecal metabolomics study by Zhao et al. [44] identified 26 metabolites progressively elevated from healthy controls to MCI and AD, among which arachidonic acid, adrenic acid, and lithocholic acid are considered critical in AD pathophysiology. Arachidonic acid, a polyunsaturated fatty acid vital to human health, is involved in oxidative metabolism, which is a hallmark of neuroinflammation, highlighting the link between lipid metabolism and AD [170,171]. Moreover, arachidonic acid mediates Aβ-induced pathogenesis, contributing to learning, memory, and behavioral deficits in AD mouse models [170]. Dysregulated metabolism of adrenic acid and arachidonic acid has been shown to trigger ferroptosis, an iron-dependent lipid peroxidation-driven form of cell death [172,173,174,175], which is increasingly recognized as a contributing mechanism in neurodegenerative diseases including AD [176]. In turn, lithocholic acid, a secondary BA synthesized in the colon from chenodeoxycholic acid by intestinal bacteria, was found to be elevated in AD patients relative to healthy controls [177], suggesting its potential as a biomarker for AD. Overall, accumulating evidence supports that alterations in BA profiles are associated with cognitive impairment in AD [167,178].

Emerging evidence also suggests that certain BAs may possess neuroprotective properties. A recent systematic review concluded that ursodeoxycholic acid (UDCA) can reduce reactive oxygen species (ROS), tumor necrosis factor-alpha (TNFα), and interleukin-1 beta (IL-1β), thereby exerting anti-apoptotic, antioxidant, and anti-inflammatory effects in AD [179]. In addition, tauroursodeoxycholic acid (TUDCA) has been demonstrated to reduce Aβ deposition, inhibit amyloid pathology progression, and suppress glycogen synthase kinase 3 beta (GSK3β) activity, which consequently reduces tau hyperphosphorylation and microglial activation [114]. Further studies have shown that TUDCA binds to the G protein-coupled bile acid receptor 1 (GPBAR1), also known as Takeda G protein-coupled receptor 5 (TGR5), on microglial cells. This interaction elevates intracellular cyclic AMP (cAMP) levels, promotes an anti-inflammatory microglial phenotype, and attenuates neuroinflammatory responses [180]. These findings highlight the potential therapeutic effects of TUDCA in the context of AD [114,181].

Several anaerobic bacteria within the GM, including members of the *Clostridiaceae* and *Enterobacteriaceae* families, have been shown to metabolize dietary substrates such as carnitine, choline, and lecithin into trimethylamine (TMA). TMA is subsequently absorbed and oxidized in the liver to form trimethylamine *N*-oxide (TMAO) [134]. Elevated levels of TMAO have been detected in the CSF of patients with MCI and AD, correlating with pathological hallmarks of AD, including tau phosphorylation, Aβ deposition, and neurodegeneration [135]. Mechanistically, TMAO activates the NOD-like receptor protein 3 (NLRP3) inflammasome, thereby triggering inflammatory responses via the SIRT3-SOD2-mitochondrial reactive oxygen species (mtROS) pathway [182]. Moreover, TMAO adversely affects the tricarboxylic acid (TCA) cycle by reducing ketone and fatty acid oxidation, leading to impaired energy metabolism and mitochondrial dysfunction. This metabolic disruption promotes downregulation of synaptic plasticity-related proteins and suppression of the mTOR signaling pathway, resulting in mitochondrial damage, increased superoxide production, synaptic impairment, and cognitive decline [183]. Notably, interventions that decrease plasma TMAO levels have been shown to reduce pro-inflammatory cytokines such as IL-2, IL-17, and TNF-α, concomitantly improving cognitive function and slowing pathological progression [184].

### 4.3. Tryptophan Metabolites

Trp is an essential aromatic amino acid obtained through dietary intake that is subject to regulation by the GM [185]. Recent metabolomics analyses have revealed significant differences in Trp-derived catabolites (TRYCATs) between individuals with AD and healthy controls, suggesting that dysregulated Trp metabolism, which is driven by microbial imbalance, may contribute to cognitive impairment in AD [47]. A key pathway implicated in this dysregulation is the kynurenine pathway (KP), which appears to play a critical role in AD pathophysiology through the exacerbation of neuroinflammation [186]. The GM has been shown to influence circulating Trp levels, directing its metabolism through the KP and resulting in the production of several neuroactive metabolites. Among these, there are neuroprotective compounds such as KYN and the antioxidant 3-hydroxyanthranilic acid (3-HAA), as well as neurotoxic metabolites including 3-hydroxykynurenine (3-HK) and quinolinic acid (QUIN) [187]. In the context of AD, KP dysregulation is characterized by: (i) reduced plasma concentrations of Trp and 3-HAA; (ii) elevated KYN/Trp ratios and increased serum levels of 3-HK; and (iii) accumulation of QUIN in the hippocampus [186]. Importantly, these KP-derived metabolites exhibit strong correlations with key pathological markers of AD, including amyloid-β42 (Aβ42) and phosphorylated tau (p-Tau181) [188].

Tryptophan catabolites (TRYCATs) have been shown to promote neurogenesis in murine models via mechanisms dependent on activation of the aryl hydrocarbon receptor (AhR) [189]. Notably, AhR has also been implicated in the regulation of sphingolipid metabolism, where it enhances sphingolipid and sphingosine-1-phosphate (S1P) levels, thereby contributing to the maintenance of axonal myelination and neural integrity [190]. Among Trp-derived microbial metabolites, indole plays a pivotal neuroprotective role in the context of AD. It modulates neuroinflammation, supports intestinal barrier function, and contributes to immune homeostasis [191]. Importantly, a marked reduction in the abundance of indole-producing bacteria, particularly within the phyla Actinomycetota, Bacillota, and Bacteroidota, has been reported in patients with AD, paralleled by a significant decline in circulating indole levels [47]. Indole-3-propionic acid (IPA), a specific indole derivative synthesized by *Clostridium sporogenes*, is capable of intestinal absorption and subsequent penetration of the BBB. Within the CNS, IPA exerts antioxidant effects by neutralizing hydroxyl radicals, mitigating DNA damage, and inhibiting amyloid fibril formation [191]. The neuroprotective potential of indole compounds is attributed to their multifaceted biological activities, including antioxidative, anti-inflammatory, immunomodulatory, and anti-amyloidogenic actions.

### 4.4. Neurotransmitters

Amino acids serve as essential precursors in the biosynthesis of various neurotransmitters. Increasing evidence indicates that the GM not only modulates the availability of these amino acids but also significantly influences neurotransmitter synthesis and activity [192]. Evidence has shown that gut microorganisms possess the capacity to synthesize and regulate a wide range of neuroactive compounds within the host, including GABA, serotonin, dopamine, and norepinephrine [193]. These microbiota-derived neuromodulators exert systemic effects through the MGB axis, thereby modulating neuronal metabolism and CNS function.

#### 4.4.1. Gamma-Amino Butyric Acid

GABA constitutes the principal inhibitory neurotransmitter in the CNS and plays a pivotal role in regulating neuronal excitability [194]. Dysregulation of GABA metabolism has been implicated in cognitive and memory impairments and is associated with brain-related conditions such as anxiety, depression, and AD [195]. Postmortem analyses have revealed a downregulation of GABAergic signaling components in the middle temporal gyrus of individuals with AD, along with decreased GABA levels in the CSF [196].

Emerging evidence suggests that GABA is also synthesized by the GM, with key contributions from genera such as *Alistipes*, *Bacteroides*, *Bifidobacterium*, *Blautia*, *Escherichia*, *Lacticigenium*, and *Lactobacillus* [119,120,197]. These microbial taxa harbor genes that encode glutamate decarboxylase (GAD), the enzyme responsible for converting glutamate into GABA [198,199]. In this respect, preclinical studies have shown that germ-free mice exhibit significantly lower GABA concentrations in the colon [200], suggesting that the GM plays a key role in regulating host GABA levels.

While dietary glutamate is unable to cross BBB, microbial-derived GABA has been shown to penetrate the BBB and modulate neuronal and synaptic functions [201]. Although these findings point to the GM as a potential modulator of GABAergic signaling in the CNS, the underlying mechanisms still require further elucidation. In the context of AD, GABA produced in the gut may exert its effects via multiple mechanisms. One proposed route involves the stimulation of the vagus nerve, transmitting afferent signals from the gut to the brain. Alternatively, GABA may influence the integrity of the gut mucosal barrier by modulating mucin production [120]. The mucus layer in the gastrointestinal tract serves as a critical barrier separating the GM from epithelial cells, facilitating the controlled movement of solutes in and out of the gut lumen [202]. A reduction in mucus thickness brings microorganisms and their metabolites into closer proximity with the gut epithelium, potentially disrupting tight junctions and allowing microbial products to penetrate the lamina propria [203,204]. Endogenously produced GABA in the gut may traverse the intestinal epithelium, enter the lamina propria, and subsequently reach the brain via systemic circulation [205]. Research indicates that the intestinal barrier is often compromised in individuals with AD [204], increasing the likelihood of microbial metabolites and byproducts entering the bloodstream [120,206]. Furthermore, dysregulation of tight junction proteins can facilitate the systemic translocation of both host- and microorganism-derived metabolites. Once in circulation, these molecules may cross BBB, which is also impaired in AD, potentially exacerbating neuroinflammation and neurodegeneration [207].

GABA plays a crucial role in numerous aspects of neuronal development, including synaptogenesis and the regulation of neuroinflammation [208]. Notably, a reduction in GABAB receptor expression on glial cells has been linked to elevated levels of Aβ 1–40 in transgenic mouse models [121]. Astrocytes also appear to be key mediators in the GABA metabolic cycle. Impaired astrocytic function has been shown to diminish glutamine synthesis, thereby limiting the availability of glutamine required for neuronal GABA production. This deficit contributes to an imbalance in synaptic excitation and inhibition observed in the AD brain [209]. According to Bi et al. [210], disruption of GABAergic signaling induces a shift in the excitatory/inhibitory balance, which facilitates the spread of amyloid and tau pathologies and accelerates cognitive decline in AD. However, contrasting evidence suggests that pharmacological inhibition of GABA signaling may, in certain contexts, mitigate cognitive impairment [211]. Interestingly, despite advanced neuropathology in late-stage AD, the expression levels of glutamate and GABA receptor subunits remain relatively preserved, indicating that the brain may attempt to maintain excitatory/inhibitory homeostasis even during disease progression [196]. These findings collectively underscore the GABAergic system as a promising therapeutic target for restoring neurochemical balance and cognitive function in AD.

#### 4.4.2. Serotonin

Beyond the hallmark pathologies of Aβ and tau, AD is also characterized by alterations in the serotonergic system, which plays a pivotal role in modulating both emotional and cognitive functions [122]. Enterochromaffin cells synthesize over 90% of total serotonin in the intestinal epithelium. However, the GM contributes significantly to serotonergic homeostasis, with strains of the genera *Bifidobacterium*, *Lacticigenium*, and *Roseburia* affecting it via metabolism of dietary Trp [124]. Although emerging evidence points to a role for gut-derived serotonin in CNS function, the mechanistic pathways through which this influence is exerted remain underexplored.

Selective serotonin reuptake inhibitors (SSRIs), which are widely used as antidepressants, elevate extracellular serotonin concentrations by preventing its reuptake at synaptic junctions [212]. Preclinical investigations have shown that SSRIs can improve cognitive performance in APP transgenic mice, potentially by enhancing mitochondrial biogenesis and promoting autophagic processes. Additional studies report increased dendritic spine density and synaptic activity following SSRI administration in these models [213]. In vitro data further support these findings, revealing that SSRIs reduce phosphorylated tau and serotonin protein levels, augment mitochondrial respiration, and improve neuronal cell survival [214]. However, more recent clinicopathological findings have raised concerns, suggesting that SSRIs may exacerbate cognitive decline by contributing to increased tau tangle formation [123]. These conflicting results underscore the complexity of serotonergic modulation and highlight the need for further investigation into the long-term effects of SSRIs in this context.

The precise regulation of serotonin receptor activity is essential for maintaining cognitive and mnemonic functions in individuals with AD. Disruptions in serotonergic signaling have been specifically observed within the hippocampus of AD mouse models, reinforcing the crucial involvement of serotonin in AD pathology [215]. Notably, activation of the serotonin receptor subtype 5-HT7R has been shown to exacerbate tau pathology by enhancing tau phosphorylation and aggregation through cyclin-dependent kinase 5 (CDK5) activity. This cascade contributes to neuronal degeneration, impaired long-term potentiation, and deficits in memory performance in transgenic mouse models [216]. Consistent findings by Wu et al. [115] further demonstrate that downregulation of 5-HT1A and 5-HT2A receptors enhances neuronal resilience to Aβ-induced neurotoxicity and mitigates cognitive impairments. Importantly, alterations in the GM have been shown to modulate both Trp availability and peripheral serotonin levels, ultimately influencing the central synthesis of key neurotransmitters [75]. Collectively, these findings underscore a mechanistic link between GM-mediated serotonergic regulation and the neurodegenerative processes underlying AD, highlighting serotonin signaling as a potentially modifiable contributor to AD pathogenesis.

#### 4.4.3. Acetylcholine

ACh is an excitatory neurotransmitter that is present in both the peripheral nervous system and CNS. Its synthesis and release are regulated by the central cholinergic system, which significantly influences ACh availability [217]. The transport of ACh into synaptic vesicles is mediated by two key proteins: the high-affinity choline transporter (CHT) and the vesicular acetylcholine transporter (VAChT). Impairments in ACh regulation at both presynaptic and postsynaptic sites have been recognized as contributing risk factors for AD [218].

The cholinergic system, including ACh, its transporters, and associated receptors, plays a pivotal role in cognitive processes such as learning, memory consolidation, and attentional control. Of particular importance are the basal forebrain cholinergic neurons (BFCNs), which project to the hippocampus and cortex and are central to memory and cognitive regulation [125]. In individuals with AD, there is consistent evidence of early and progressive degeneration of BFCNs, with the severity of cognitive impairment correlating with synaptic loss in their target regions [219]. Notably, the integrity of the BFC system has been proposed as a biomarker for predicting Aβ burden in AD [220], a finding that aligns with the observed relationship between hippocampal atrophy and amyloid deposition [221].

Clinical observations have confirmed pronounced neurodegeneration within the cholinergic system in AD, including marked reductions in ACh levels, loss of cholinergic neurons, and diminished choline acetyltransferase activity [125,222]. In addition, aberrant alterations in central cholinergic signaling have been implicated in pathological mechanisms such as tau hyperphosphorylation, neuroinflammation, apoptosis, and disruptions in both neurotransmitter and neurohormonal systems. However, the precise mechanistic pathways underlying these effects remain incompletely understood [223].

ACh receptors are broadly classified into two major types: muscarinic and nicotinic receptors. Muscarinic ACh receptors (mAChRs), which belong to the class I G protein-coupled receptor (GPCR) family, have been implicated in the pathophysiology of various neurological disorders, including AD. Alterations in the expression and function of mAChRs have been observed in AD and are considered potential therapeutic targets for intervention [224]. In contrast, nicotinic ACh receptors (nAChRs) are ligand-gated ion channels that mediate rapid synaptic transmission in both the peripheral nervous system and CNS. These receptors are composed of diverse subunits that combine to form multiple nAChR subtypes with distinct functional profiles [225]. Notably, nAChRs are highly expressed in brain regions that are critical for memory and cognition, such as the frontal cortex, hippocampus, substantia nigra, and thalamus [223,226].

Several gut microorganisms, including *B. subtilis*, *E. coli*, *Lactiplantibacillus plantarum*, and *Staphylococcus aureus*, are capable of producing ACh. Since ACh cannot directly cross the BBB, choline from the peripheral nervous system must be transported across the BBB via specific carrier proteins, where it is subsequently synthesized into ACh within the CNS by the enzyme choline acetyltransferase [227]. Studies have reported a reduction in choline acetyltransferase activity across all cortical regions in individuals with AD, suggesting that diminished ACh levels in memory-associated areas such as the prefrontal cortex may contribute to disease progression [228]. Moreover, impaired cholinergic neurotransmission has been closely linked to AD pathology. For instance, disruptions in cortical cholinergic signaling can alter the expression and processing of APP, promoting the deposition of Aβ plaques, which in turn exacerbate cholinergic dysfunction [229].

#### 4.4.4. Dopamine

Dopamine is a pivotal catecholamine neurotransmitter in mammals that influences both the peripheral nervous system and the CNS [230]. Its established roles in the prefrontal cortex include regulating emotional processing, maintaining and manipulating working memory, and facilitating motor command transmission [231]. Beyond its well-known involvement in reward and motivation, dopamine systems also modulate brain regions involved in chronic pain [232]. The activity of monoamine oxidase (MAO), an enzyme responsible for dopamine metabolism, influences neurotransmitter concentrations, with alterations linked to several neurological disorders, including AD and Parkinson’s disease. Notably, MAO inhibition has been shown to exert neuroprotective effects by reducing oxidative stress mediated by MAO enzymes, suggesting a potential therapeutic avenue in AD [233]. Compared to healthy controls, AD patients display significantly reduced levels of dopamine as well as dopamine receptor subtypes D1 and D2, indicating a potential relationship between dopaminergic dysfunction and AD progression [234]. Furthermore, degeneration of dopaminergic neurons in the ventral tegmental area correlates with memory deficits and impaired consciousness in AD [127]. Early pathological features such as tau neurofibrillary tangles and Aβ deposition deficiencies in the hippocampus of the 3xTg-AD mouse model further contribute to dopaminergic dysfunction [235].

The GM has been identified as a significant source of dopamine, with genera such as *Bacillus*, *Bacteroides*, *Bifidobacterium*, and *Brevilactibacter* playing predominant roles in its synthesis and bioavailability. Dopamine produced by the GM is metabolized and transported across the BBB to the brain, where it exerts an inhibitory effect on the NLRP3 inflammasome via dopamine receptors expressed on microglia and astrocytes, thereby modulating neuroinflammatory responses [128]. The D4 dopamine receptor specifically regulates the trafficking of α-amino-3-hydroxy-5-methyl-4-isoxazole-propionic acid (AMPA) receptors in GABAergic interneurons within the prefrontal cortex through a distinctive signaling pathway, influencing the strength of excitatory synapses and affecting cognition and emotion [236]. In AD model rats, degeneration of midbrain limbic dopamine neurons impairs the function of parvalbumin-positive interneurons [237], leading to early hippocampal hyperexcitability and epilepsy-like activity [129], a phenomenon that contrasts with the role of ACh in AD pathology.

#### 4.4.5. Norepinephrine

As previously stated, norepinephrine is another catecholamine that can be produced by specific gut microorganisms [238]. While norepinephrine is primarily synthesized by noradrenergic neurons in the LC of the brainstem, certain gut bacteria, including *B. subtilis*, *E. coli*, and *Proteus vulgaris*, also produce this neurotransmitter [130]. The depletion of LC neurons in the early stages of AD has been well-documented. In transgenic AD mouse models, destruction of LC neurons prior to amyloid plaque formation significantly worsens cognitive impairment [239]. In this respect, norepinephrine plays a critical role in cognitive functions, including cognitive flexibility and active memory processes [240].

According to existing literature, norepinephrine has been shown to modulate synaptic plasticity [241], regulate the activation of microglia and astrocytes, and enhance the production of brain-derived neurotrophic factor (BDNF), thereby exerting neuroprotective effects [242]. These functions are pivotal for maintaining brain health, especially in the context of neurodegenerative diseases such as AD. Postmortem CSF samples from AD patients have revealed reduced levels of norepinephrine and its metabolites [243], providing evidence of the involvement of norepinephrine in AD pathogenesis. Studies in APP/PS1 transgenic mice have demonstrated a strong correlation between neuroinflammation, microglial activation, and degeneration of the LC-norepinephrine system [244]. In addition, deficiencies in this system have been linked to Aβ deposition [245], tau protein hyperphosphorylation [246], and impairments in spatial memory [247,248]. The LC-norepinephrine system is thus postulated to play a critical role in AD pathology by reducing the release of inflammatory cytokines and promoting amyloid clearance via regulation of microglial migration and phagocytosis [249,250].

## 5. Therapeutic Approaches Targeting the Gut Microbiome

In light of its widespread prevalence and devastating impact on cognitive function, AD is recognized as the most prevalent form of dementia and one of the most prominent contributors to mortality globally. Currently, therapeutic options for AD remain limited, primarily consisting of acetylcholinesterase inhibitors, anti-inflammatory agents such as curcumin, ellagic acid, and galantamine, as well as interventions aimed at reducing Aβ deposition and tau phosphorylation, including electroacupuncture, isoastilbin, and plant polyphenols. In this section, the theoretical foundations of clinical treatments and experimental research focusing on the GM as a promising therapeutic avenue are outlined. In this respect, emerging treatment strategies for AD emphasize the regulation of dysbiotic GM composition, as well as the modulation of neuroendocrine activities via metabolic signaling pathways.

### 5.1. Probiotics

Probiotics are defined as live microbial supplements and have demonstrated efficacy in restoring GM balance and providing therapeutic benefits for patients with AD [20]. Nevertheless, evidence supporting their clinical outcomes is still limited. Recent research highlights the promising effects of a novel probiotic formulation, SLAB51, which comprises strains such as *Streptococcus thermophilus* DSM 32245, *Bifidobacterium animalis* subsp. *lactis* DSM 32246 and DSM 32247, *Lactobacillus acidophilus* DSM 32241, *Lactobacillus helveticus* DSM 32242, *Lacticaseibacillus paracasei* DSM 32243, *L. plantarum* DSM 32244, and *Levilactobacillus brevis* DSM 27961. This probiotic cocktail has been shown to improve glucose homeostasis, reduce tau phosphorylation [251], and increase SCFAs such as acetate, butyrate, and propionate, while simultaneously decreasing inflammation and Aβ deposition [252]. In addition, SLAB51 activates a SIRT1-dependent pathway that mitigates oxidative stress in the brains of AD mouse models [253]. More recently, SLAB51 has been reported to inhibit cholesterol biosynthesis, reduce the ω-6/ω-3 fatty acid ratio, and attenuate neuroinflammation and oxidative stress, collectively leading to decreased aggregation of Aβ and tau proteins and slowing AD progression [254].

As demonstrated by Song et al. [255], the probiotic *L. plantarum* strain DP189 effectively regulates GM dysbiosis and inhibits tau hyperphosphorylation. Furthermore, this strain has been shown to suppress the production of trimethylamine (TMA) and its hepatic metabolite trimethylamine-*N*-oxide (TMAO), leading to reduced expression of the *clu* gene (clusterin). These effects collectively alleviate neuroinflammation and neuropathological defects in APP/PS1 transgenic mice [256].

The probiotic cocktail VSL#3, comprising eight strains, *L. plantarum*, *Lactobacillus delbrueckii* subsp. *bulgaricus*, *L. acidophilus*, *L. paracasei*, *Bifidobacterium breve*, *Bifidobacterium longum*, *B. longum subsp*. *infantis*, and *Streptococcus salivarius* subsp. *thermophilus*, has been shown to effectively reduce serum levels of prostaglandin and deoxycholic acid. This reduction contributes to the amelioration of intestinal inflammation and gut permeability. However, its effects on brain amyloid plaque deposition, pro-inflammatory cytokine levels, and gliosis appear to be limited [257].

In summary, probiotics have been demonstrated to modulate GM composition and lipid metabolism homeostasis, thereby exerting beneficial effects on brain inflammation, oxidative stress, and the pathologies of Aβ and tau. Thus, targeting the microbial community through probiotic interventions offers promising avenues for the development and clinical evaluation of novel preventive and therapeutic strategies for AD.

### 5.2. Prebiotics

Prebiotics, comprising human milk oligosaccharides, non-digestible oligosaccharides, and soluble fermentable fibers, represent a promising alternative or complement to probiotic supplementation. These compounds have been shown to enhance the growth of beneficial bacteria, such as bifidobacteria and lactobacilli, thereby improving cognitive impairment in APP/PS1 mice via the MGB axis [258,259]. The neuroprotective properties of prebiotics highlight their potential as oral formulations for the prevention and treatment of AD [260]. Specifically, fructooligosaccharides have demonstrated beneficial effects in APP/PS1 mice [261], while mannanoligosaccharides have been found to reshape the GM, maintain intestinal barrier integrity, increase SCFA production, inhibit neuroinflammation and oxidative stress, and alleviate cognitive and behavioral deficits in 5xFAD mice [262]. In addition, xylooligosaccharides, abundant plant-derived biopolymers resembling oligosaccharides, have been shown to significantly alter GM composition and improve AD symptoms such as anxiety and depression, and may also help preserve or enhance cognitive function in individuals at risk for AD. [262,263]. While prebiotic supplementation holds promise for modulating microbial composition and function to improve AD outcomes, further research is necessary to fully assess their therapeutic applicability.

### 5.3. Fecal Microbiota Transplantation

Fecal microbiota transplantation (FMT) involves transferring the fecal microbiota of a healthy donor into the gut of a patient or diseased animal to restore GM balance and improve disease outcomes [264]. Increasing evidence supports the therapeutic potential of FMT from wild-type (WT) mice in ameliorating AD-related pathologies in transgenic ADLPAPT mouse models. This intervention has been shown to reduce Aβ plaques, neurofibrillary tangles, glial reactivity, and cognitive impairments, indicating that restoring GM homeostasis via FMT may benefit AD treatment [265]. Similarly, FMT from WT mice has been reported to positively reshape the GM in APP/PS1 mice by increasing Bacteroidota abundance while reducing Pseudomonadota and Verrucomicrobiota populations, elevating butyrate levels, and significantly improving Aβ accumulation, synaptic dysfunction, neuroinflammation, and cognitive deficits [266]. Moreover, Qian et al. [267] demonstrated that FMT from WT mice modulates glycerophospholipid metabolism in APP/PS1 mice, further alleviating Aβ pathology and neuroinflammation. In contrast, FMT from AD mice has been found to impair memory and neurogenesis in WT mice [268,269]. Nevertheless, the precise mechanisms by which GM influences these outcomes require further clarification, but these findings collectively suggest a promising role for healthy FMT in modulating AD pathology [270].

A clinical trial has reported that FMT improved clinical manifestations in AD patients, notably cognitive impairments. For instance, a case study described an AD patient who received FMT from a healthy 47-year-old woman and subsequently exhibited significant improvements in cognitive function, as evidenced by enhanced mini-mental state examination and clinical dementia rating scores [271]. Despite the promising efficacy and safety of FMT demonstrated in murine models, several challenges remain for its clinical application. These include acceptance and compatibility between recipients and donors, individual variations in immune responses, and lifestyle factors, such as diet, that influence GM composition. Furthermore, relatively few studies have systematically addressed GM variations following FMT. Table 3 provides an overview of current research on GM-based therapies aimed at mitigating AD-related pathological features.

## 6. Discussion

This review was aimed at summarizing current knowledge on GM dysbiosis in AD, with special attention to microbial metabolomes and GM-targeted therapeutics. Based on the reviewed literature, several key points can be outlined: (i) AD is linked to reduced gut microbial diversity and altered bacterial taxa, varying by region and disease stage; (ii) GM shifts correlate with inflammation and may drive AD progression via the MGB axis; (iii) specific gut microorganisms correlate with AD biomarkers, linking microbial alterations to amyloid and tau pathology; (iv) GM profiles differ between AD, MCI, and controls, with shifts in key bacterial genera associated with disease progression and cognitive decline; (v) microbial amyloids and bacterial products (e.g., LPS) can cross the intestinal barrier and BBB, triggering neuroinflammation and promoting amyloid and tau pathologies in AD; (vi) GM dysbiosis disrupts the gut-immune barrier and exacerbates systemic inflammation, contributing to AD pathogenesis via immune and molecular mechanisms; (vii) reduced sphingolipid and phospholipid levels are linked to AD progression, correlating with amyloid deposition, brain atrophy, and cognitive decline; (viii) LPS from Gram-negative bacteria contribute to AD by disrupting barriers, promoting neuroinflammation, Aβ deposition, and tau hyperphosphorylation; (ix) SCFAs produced by the GM regulate neuroinflammation, lipid metabolism, and gene expression via GPCR signaling, impacting AD pathology; (x) butyrate and acetate improve cognition by epigenetic modulation (i.e., HDAC inhibition), enhancing synaptic plasticity and reducing pro-inflammatory cytokines through multiple pathways; (xi) SCFAs cross the BBB to promote neuronal repair, suppress microglial and astrocyte inflammation, and modulate amyloid and tau pathology, helping mitigate cognitive decline in AD; (xii) AD is linked to increased secondary BAs and decreased primary BAs, promoting amyloid pathology and cognitive decline; (xiii) UDCA and TUDCA show neuroprotection by reducing inflammation, Aβ, and tau pathology in AD; (xiv) dysregulated Trp metabolism via the KP, driven by GM imbalance, contributes to AD neuroinflammation and correlates with amyloid and tau pathology; (xv) microbial-derived indole metabolites, especially indole-3-propionic acid, have neuroprotective effects by reducing oxidative stress, neuroinflammation, and amyloid formation in AD; (xvi) the GM synthesize GABA, which crosses the BBB to modulate brain function and may affect AD via vagus nerve or gut barrier pathways; (xvii) AD involves GABAergic dysfunction causing excitatory/inhibitory imbalance and neuroinflammation, making GABA signaling a therapeutic target; (xviii) the GM produce the majority of serotonin in the body from dietary Trp, impacting CNS function and serotonergic dysregulation in AD; (xix) serotonin receptor changes and SSRIs have mixed effects on tau pathology and cognition in AD models, highlighting complex roles and therapeutic potential (xx) cholinergic system dysfunction, including loss of basal forebrain cholinergic neurons and reduced ACh synthesis, correlates with cognitive decline and AD pathology; (xxi) gut microorganisms produce ACh precursors, but impaired choline transport and cholinergic signaling disruption contribute to Aβ accumulation and AD progression; (xxii) dopaminergic dysfunction in AD involves reduced dopamine levels, receptor loss, and neuron degeneration, contributing to memory deficits and neuroinflammation; (xxiii) the GM produces dopamine that modulates brain inflammation via microglial receptors and influences synaptic function, linking gut–brain interactions to AD pathology; (xxiv) norepinephrine, which is produced by LC neurons and certain gut bacteria, is crucial for cognitive function and is depleted early in AD, worsening cognitive decline; (xxv) norepinephrine modulates synaptic plasticity, regulates microglial and astrocyte activation, enhances BDNF production, and promotes amyloid clearance, with LC-norepinephrine system dysfunction linked to AD pathology and memory impairments; (xxvi) probiotic formulations such as SLAB51 and *L. plantarum* DP189 modulate the GM and also reduce tau phosphorylation, Aβ deposition, neuroinflammation, and oxidative stress, improving AD pathology and slowing disease progression; (xxvii) probiotics influence lipid metabolism and gut barrier integrity, with some reducing intestinal inflammation and metabolites linked to AD, supporting their potential as therapeutic agents for AD; (xxviii) prebiotics promote beneficial gut bacteria growth, improving cognition and reducing neuroinflammation in AD mouse models via the MGB axis; (xxix) specific prebiotics such as fructooligosaccharides and mannanoligosaccharides enhance gut barrier integrity, increase SCFAs, and potentially alleviate AD symptoms; (xxx) FMT from healthy donors has demonstrated to restore GM balance, reducing Aβ plaques, neuroinflammation, and cognitive deficits in AD mouse models; (xxxi) clinical evidence suggests FMT can improve cognitive function in AD patients, although challenges remain in donor–recipient compatibility and understanding mechanisms.

AD is a prevalent neurodegenerative disorder characterized by hallmark neuropathological features, including extensive extracellular Aβ plaque formation and the intracellular accumulation of hyperphosphorylated tau fibrils. These pathological changes are accompanied by neuroglial proliferation and progressive neuronal loss. The GM plays a pivotal role in maintaining overall host health, including brain function. However, the development of GM-targeted therapies depends on a comprehensive understanding of the GM ecosystem and the complex interactions between microbial metabolites and the host throughout the human lifespan [281]. To determine whether microorganisms are causative agents in AD pathogenesis or simply reflect homeostatic dysregulation secondary to the disease, longitudinal clinical studies are essential. Such studies would clarify the temporal relationship between GM dysbiosis and the onset of AD [282]. This task is complicated by the fact that AD pathology begins approximately 20 years before clinical symptoms emerge, making it difficult to establish causality and define the optimal therapeutic stage [283,284]. Nevertheless, assessing GM biomarkers at presymptomatic stages could reveal early disruptions in gut homeostasis, along with altered metabolite and neuroactive compound profiles, which may serve as prodromal indicators of AD [285,286]. Advancements in technologies such as high-throughput sequencing and next-generation meta-omics are poised to provide deeper insights into the gut environment of individuals suffering from cognitive impairments and neurodegenerative diseases such as AD [287]. Interventions encompassing cognitive stimulation, dietary modification, physical activity, and social support hold promise as both preventative and therapeutic approaches to mitigate cognitive decline. However, optimizing these strategies is essential to ensure maximal benefits for older adults. In recent years, increasing emphasis has been placed on promoting healthy aging, including the prevention and management of neurodegenerative diseases. Although interventions aimed at supporting healthy aging encompass multiple health domains, they are often heterogeneous, raising concerns regarding their applicability across different settings, reproducibility, and overall efficacy [288]. Among these, cognitive stimulation has emerged as a noteworthy approach, expanding into diverse healthcare areas and becoming a broadly interdisciplinary tool in the care of older adults. Despite its apparent potential, most studies assessing its effectiveness exhibit methodological biases that undermine the validity of their findings [289]. Therefore, it is essential that interventions targeting adverse health outcomes associated with aging, such as AD, undergo continuous refinement to achieve more conclusive evidence regarding their efficacy. This undoubtedly requires the concurrent integration of emerging strategies, such as microbial-based approaches, which themselves demand a deeper understanding of the underlying mechanisms involved.

Given the close interplay between the GM and brain-related disorders such as AD, coupled with the current lack of effective treatments for this specific neurodegenerative condition, novel therapeutic approaches targeting microorganisms and their metabolites have emerged. These include the use of psychobiotics, FMT, and cutting-edge biotechnologies such as phage therapy, microbial encapsulation, and bioengineered microorganisms capable of producing neuroprotective metabolites [33,290,291]. Microbial-based treatments may reduce Aβ deposition by restoring GM homeostasis and lowering the production of inflammatory factors, thereby potentially improving cognitive function. However, further efforts are required to develop effective and well-tolerated microbial therapies suitable for clinical application [287]. Although preclinical studies have built the foundation for these approaches, fully transitioning to clinical trials demands a deeper understanding of the underlying mechanisms and the creation of reliable strategies for their practical implementation in humans. As these challenges are progressively addressed, more sophisticated clinical evaluations are likely to become feasible. In addition, recent advances in the development of allosteric modulators, covalent inhibitors, dual-target inhibitors, proteolysis-targeting chimeras (PROTACs), protein–protein interaction modulators, and selective inhibitors are anticipated to provide valuable insights that will inform the design and clinical translation of innovative therapies for AD [292].

It is important to acknowledge that older adults have been disproportionately affected by a range of recent global stressors, including healthcare fragmentation, increased social isolation, and reduced access to preventive and cognitive health services. These circumstances have intensified age-related vulnerabilities and underscored the urgent need for targeted strategies to support healthy aging [293]. Thus, integrating biological insights into broader models of care may help refine interventions aimed at preserving function, enhancing quality of life, and addressing the multifactorial challenges faced by aging populations, particularly the prevention and management of prevalent conditions such as AD. Indeed, emerging epidemiological and clinical evidence indicates that lifestyle interventions, including diet and physical activity, may provide an alternative therapeutic approach to slow or prevent cognitive decline and dementia. For instance, Arora et al. [294] report that foods featured in Mediterranean, MIND, and DASH diets, such as fruits, leafy green vegetables, nuts, and olive oil, may help prevent or attenuate cognitive decline. Considering the predominantly plant-based foods emphasized in these diets, it is plausible that well-planned vegetarian diets may also play a promising role in gerontological contexts, given their increasing acceptance in clinical practice, their multiple health benefits, and their broader ethical and sustainability implications [295]. Nevertheless, the precise mechanisms by which the nutrients present in these diets support brain health remain incompletely understood. In addition, a pilot study showed that cognitively impaired individuals exhibited improved executive function when engaging in aerobic exercise combined with a DASH diet [296]. In this respect, combination therapies are gaining attention, either through the co-development of multiple new molecular entities or via add-on strategies pairing an approved agent with a novel compound [297]. Recently approved anti-amyloid monoclonal antibodies, including lecanemab and donanemab, have been shown to slow disease progression by approximately 30%, and combination approaches may be required to prevent AD onset or achieve greater slowing of cognitive and functional decline [298].

This narrative review may present potential limitations that should be carefully considered when interpreting the results. First, it does not include a formal quality assessment or risk-of-bias evaluation of the selected studies. Second, the heterogeneity in study designs, animal models, and human cohorts, as well as variations in GM profiling methods and geographic differences, limit the generalizability of findings. Third, the complexity of AD pathophysiology and gut–brain interactions, including multiple microbial metabolites, neurotransmitter pathways, and immune mechanisms, poses challenges in establishing direct causal relationships. Fourth, while probiotics, prebiotics, and FMT show therapeutic promise, their long-term safety, optimal formulations, and mechanisms of action require further investigation in well-controlled clinical trials. Finally, individual factors such as diet, lifestyle, and genetics, which influence GM composition and AD progression, were not systematically addressed in most of the studies reviewed, highlighting the need for personalized approaches in future research.

## 7. Conclusions

Prior studies have revealed that the GM plays a pivotal role in regulating the development of AD pathologies. Advances in research on the relationship between the GM and AD increasingly support the hypothesis that modulating the GM could become a promising strategy to slow AD progression. Importantly, microbial metabolites such as SCFAs, BAs, Trp derivatives, and neurotransmitters including GABA, serotonin, and dopamine appear to mediate key aspects of neuroinflammation, synaptic function, and amyloid or tau pathology. In addition, modulation of gut-derived signaling molecules through the MGB axis offers a complementary route to influence central processes such as neurogenesis, inflammation, and barrier integrity.

Various therapeutic interventions, including the use of probiotics, prebiotics, and FMT, require further investigation in order to elucidate their potential for restoring microbial balance and mitigating AD pathophysiology. However, the effective application of these approaches depends on a thorough understanding of the mechanistic pathways linking the GM to AD pathology, which is essential to ensure both safety and efficacy. Clinical research into GM modulation in AD remains in its early stages, and additional evidence is needed to confirm the benefits of these interventions. The field faces several challenges, such as the inherent complexity of the GM, individual variability, and the need for standardized protocols alongside rigorous study designs. To fully evaluate the long-term impacts of GM modulation on AD progression, longitudinal studies are crucial. Furthermore, the development of innovative methods for selective GM modulation, such as microbial encapsulation, phage therapy, enzyme modulators, and bioengineered microorganisms capable of producing beneficial metabolites, holds promise. These novel strategies may enable controlled-release and targeted interventions, promoting efficient restoration of GM balance and function. Thus, it is plausible to assert that, while GM modulation presents a compelling potential strategy to decelerate AD progression, more research is required to elucidate the complex interactions between the GM and AD, and also to establish effective, safe clinical interventions.

## Figures and Tables

**Table 1:** Clinical studies highlighting major differences in GM bacterial taxa abundance between AD patients and healthy controls.

Bacterial Taxa	Increased Abundance	Decreased Abundance	Characteristics of The Study	References
Phylum Acidobacteriota	X		Systematic review	[37]
Phylum Actinomycetota		X		[37]
		X	China/16S rRNA	[45]
Genus *Adlercrutzia*		X	USA/16S rRNA	[43]
Genus *Atopobium*	X		China/16S rRNA	[32]
Genus *Bifidobacterium*	X		Systematic review	[38]
	X		China/16S rRNA	[39]
	X		China/16S rRNA	[40]
		X		[43]
Phylum Bacillota		X		[37,38,45]
		X	China/16S rRNA	[41]
Family *Acidaminococcaceae*		X		[37]
Family *Clostridiaceae*		X		[38,43]
Family *Enterococcaceae*	X			[45]
Family *Gemellaceae*	X			[43]
Family *Lachnospiraceae*		X		[37,41,43,45]
Family *Lactobacillaceae*	X			[45]
Family *Mogibacteriaceae*		X		[43]
Family *Peptostreptococcaceae*		X		[43]
Family *Ruminococcaceae*	X			[37,45]
		X		[41,43]
Family *Turicibacteraceae*		X		[43]
Family *Veillonellaceae*		X		[45]
Genus *Agathobacter*	X			[32]
Genus *Anaerostipes*		X	China/16S rRNA	[44]
Genus *Bacillus*	X			[44]
Genus *Blautia*	X			[39,43]
		X		[41]
Genus *Butyricicoccus*		X		[40]
Genus *Clostridium*		X		[40,43]
Genus *Coprococcus*		X		[40]
	X			[32]
Genus *Dialister*		X		[40,43]
Genus *Dorea*	X			[39]
Genus *Erysipelatoclostridium*		X		[32]
Genus *Eubacterium*		X	MCI/Italy/qPCR	[35]
	X			[32]
Genus *Faecalibacterium*		X		[40]
	X			[32]
Genus *Gemella*	X			[43]
Genus *Lachnoclostridium*		X	USA/NextSeq500	[36]
Genus *Lactobacillus*	X			[39]
Genus *Limosilactobacillus*		X		[44]
Genus *Parvimonas*	X			[32]
Genus *Phascolarctobacterium*	X			[38,43]
Genus *Romboutsia*		X		[40]
Genus *Roseburia*		X		[40]
Genus *Ruminiclostridium*		X		[37]
Genus *Ruminococcus*	X			[45]
		X		[41]
Genus *Solobacterium*	X			[32]
Genus *Staphylococcus*	X			[44]
Genus *Streptococcus*	X			[39]
Genus *Subdoligranulum*		X		[45]
Genus *Turicibacter*		X		[43]
Genus *Tyzzerella*		X		[32]
Phylum Bacteroidota	X			[43,45]
Family *Bacteroidaceae*	X			[43]
		X		[45]
Family *Rikenellaceae*		X		[38]
	X			[43]
Genus *Alistipes*	X			[36,43]
		X		[39]
Genus *Alloprevotella*		X		[39]
	X			[32]
Genus *Bacteroides*	X			[36,37,43,45]
Genus *Barnesiella*	X			[36]
		X		[39]
Genus *Butyricimonas*		X		[39]
Genus *Odoribacter*	X			[36]
Genus *Parabacteroides*		X		[39]
Genus *Paraprevotella*		X		[39]
Genus *Prevotella*		X		[39]
		X	Meta-analysis	[42]
Phylum Pseudomonadota	X			[38,41]
Family *Enterobacteriaceae*	X			[41]
Genus *Acinetobacter*	X			[39]
Genus *Bosea*	X			[44]
Genus *Dyella*	X			[44]
Genus *Escherichia/Shigella*	X			[35]
Genus *Gemmiger*		X		[40]
Genus *Haemophilus*		X		[39,42]
Genus *Massilia*	X			[44]
Genus *Pseudomonas*	X			[32]
Genus *Sphingomonas*	X			[44]
Genus *Stenotrophomonas*	X			[44]
Genus *Succinivibrio*		X		[39]
Genus *Sutterella*		X		[39,42]
Genus *Variovorax*	X			[44]
Phylum Synergistota				
Genus *Cloacibacillus*	X			[32]
Phylum Thermodesulfobacteriota				
Genus *Bilophila*	X			[43]
Phylum Verrucomicrobiota		X		[45]
Genus *Akkermansia*	X			[39,40]

MCI: mild cognitive impairment. All studies focused on AD unless otherwise specified.

**Table 3:** Research on the microbial-based therapy in improving AD pathological features.

Intervention	Alterations in the GM	Major Effects	Reference
Probiotics			
*Clostridium butyricum* strain CGMCC 9831 (1 × 10^9^ CFU/mL; oral; 4 weeks) APP/PS1 mice	Increase *Alloprevotella* and butyrate. Decrease Deferribacterota, *Helicobacteraceae*, *Helicobacter*.	- Ameliorate microglia activation, neurodegeneration, Aβ deposition and cognitive deficits.	[161]
SLAB51 (2 × 10^11^ bacteria/kg/d); oral; 4–12 months) 3xTg-AD mice	Increase *Bifidobacterium* and SCFAs. Decrease Campylobacterales.	- Increase glucose metabolism. - Ameliorate lipid metabolism disorders. - Alleviate inflammatory response. - Reduce brain oxidative stress. - Decrease Aβ deposition. - Enhance cognition.	[251,252,253,254]
*Lactiplantibacillus plantarum* strain DP189 (1 × 10^9^ CFU/g; oral; 10 weeks) APP/PS1 mice	Decrease in TMA and TMAO. Restore GM dysbiosis.	- Alleviate neurotransmission. - Reduce hippocampal Aβ levels. - Improve cognitive. - Inhibit tau hyperphosphorylation.	[255,256]
VSL#3 (3.2 × 10^8^ CFU/25 g; oral; 8 weeks) APP NL-GF mice	Increase Actinomycetota and Verrucomicrobiota.	- Regulate inflammatory response.	[257]
*Bifidobacterium animalis* subsp. *lactis* strain Probio-M8 (1 × 10^9^ CFU/g; oral; 45 days) APP/PS1 mice	Increase *Desulfovibrionaceae*, *Coprococcus*, *Oscillospira*, Clostridiales. Decrease *Adlercreutzia*, *Lactobacillus*, *Streptococcus*.	- Reduce Aβ plaque burden. - Restore GM dysbiosis. - Alleviate cognitive impairment.	[272]
*B. longum* subsp. *infantis* strain BLI-02, *B. breve* strain Bv-889, *B. animalis* subsp. *lactis* strain CP-9, *B. bifidum* strain VDD088, and *L. plantarum* strain PL-02 (1 × 10^10^ CFU/capsule; oral; 12 weeks) Human AD patients	Increase *Akkermansia*, *Bifidobacterium*, *Clostridium*, *Lactobacillus* and *Ruminococcus*. Decrease *Megamonas*.	- Less cognitive deterioration. - Enhance BDNF. - Ameliorate inflammation and oxidative stress.	[273]
*Bifidobacterium breve* strain A1 (5 × 10^9^ CFU/mL; oral; 11 days) Aβ injection mice.	Increase Actinomycetota, *Bifidobacteriaceae*, and acetate. Decrease *Lachnospiraceae* and *Odoribacteraceae*.	- Suppress inflammation and immune response. - Enhance cognitive and exocrine secretion. - Inhibit gut motility.	[274]
*Bifidobacterium longum* strain NK46 (1 × 10^9^ CFU/mouse/day; oral; 2 months) 5xFAD-Tg mice	Increase *Prevotellaceae*. Decrease *Lachnospiraceae*, *Helicobacteraceae*, *Pseudomonadaceae*, *Ruminococcaceae*, and LPS.	- Inhibit inflammatory responses. - Reduce Aβ production and accumulation. - Alleviate memory and cognitive decline.	[275]
*Limosilactobacillus fermentum* strain CGMCC 18206, *L. fermentum* strain CGMCC 18207, *Lactiplantibacillus plantarum* strain CGMCC 18208, and *L. plantarum* CGMCC 18209 (1 × 10^9^ CFU/mL; oral; 12 weeks) APP/PS1 mice	Increase Bacteriodota, *Staphylococcus*, *Acinetobacter*, *Butyricicoccus*, *Sphingobacterium*, *Weissella*. Decrease Pseudomonadota, Desulfobacterota, Patescibacteria, *Eisenbergiella*.	- Reduce oxidative stress. - Attenuate microglia activation. - Improve cognitive deficiencies.	[276]
Prebiotics			
Mannanoligosaccharides (0.12%, *w*/*v* in the drinking water; oral; 8 weeks) 5xFAD-Tg mice	Increase *Lactobacillus*, *Oscillospira*, *Prevotella* and butyrate. Decrease *Helicobacter* and LPS.	- Ameliorate microglia activation, neurodegeneration, Aβ deposition and cognitive deficits.	[262]
Xylooligosaccharides (10%, *w*/*v* in PBS; oral; 5 weeks) APP/PS1 mice	Increase *Bifidobacterium*, *Lactobacillus* and *Muribacterium.*	- Decrease pro-inflammatory cytokine levels. - Normalization of microglia inflammatory response	[263]
*Morinda officinalis* oligosaccharides (50–100 mg/kg/d; 6 months) APP/PS1 mice	Increase *Akkermansia*, *Allobaculum, Arthrobacter*, *Bifidobacterium*, *Brevilactibacter*.	- Reduce neuronal apoptosis. - Improve memory.	[277]
FMT preclinical trials			
Donor: WT mice; Recipient: APP/PS1 mice	Increase Bacteroidota and butyrate. Decrease Pseudomonadota and Verrucomicrobiota.	- Alleviate cognitive deficits and neuroinflammation. - Reduce Aβ accumulation and synaptic dysfunction.	[266]
Donor: C57BL/6 mice; Recipient: C57BL/6 mice treated with antibiotics	Reduction in SCFA-producers bacteria	- Impair spatial learning and memory. - Acquisition of an aging-like phenotype of microglia cell.	[278]
Donor: B6S52 mice; Recipient: 5xFAD-Tg mice	Increase GM composition and SCFAs.	- Increase clearance of cortical Aβ. - Decrease amyloid burden and amyloid plaque. - Improve spatial memory levels.	[279]
FMT clinical trials			
Donor: 27-year-old healthy man; Recipient: 90-year-old woman with AD	Increase α-diversity and SCFAs	- Improve cognitive functions.	[271]
Donor: healthy man; Recipient: patients diagnosed with dementia with relapsed *Clostridioides difficile* infection	Increase the enrichment of Pseudomonadota and Bacteroidota	- Improve cognitive functions. - Increase in the expression of three genes associated with lipid metabolism.	[280]

SCFAs: short-chain fatty acids; FMT: fecal microbiota transplantation; BDNF: brain-derived neurotrophic factor; WT: wild-type; GM: gut microbiome.

## Abbreviations

The following abbreviations are used in this manuscript:

Aβ	Amyloid-beta
ACh	Acetylcholine
AD	Alzheimer’s disease
AhR	Aryl hydrocarbon receptor
APP	Amyloid precursor protein
BAs	Bile acids
BBB	Blood–brain barrier
BDNF	Brain-derived neurotrophic factor
BFCNs	Basal forebrain cholinergic neurons
CNS	Central nervous system
CSF	Cerebrospinal fluid
FMT	Fecal microbiota transplantation
GABA	γ-aminobutyric acid
GAD	Glutamate decarboxylase
GM	Gut microbiome
GPRs	G protein-coupled receptors
3-HAA	Antioxidant 3-hydroxyanthranilic acid
HDACs	Histone deacetylases
3-HK	3-hydroxykynurenine
HPA	Hypothalamic–pituitary–adrenal
KP	Kynurenine pathway
KYN	Kynurenic acid
LC	Locus coeruleus
LPS	Lipopolysaccaharides
mAChR	Muscarinic acetylcholine receptors
MAO	Monoamine oxidase
MAPK	Mitogen-activated protein kinase
MCI	Mild cognitive impairment
MGB	Microbiota–gut–brain
MYD88	Myeloid differentiation primary response 88
nAChR	Nicotinic acetylcholine receptors
NF-κB	Nuclear factor-κB
NLRP3	NOD-like receptor protein 3
NOX2	NADPH oxidase 2
p-tau	Phosphorylated tau
QUIN	Quinolinic acid
SCFAs	Short-chain fatty acids
SSRIs	Selective serotonin reuptake inhibitors
TLR4	Toll-like receptor 4
TMA	Trimethylamine
TMAO	Trimethylamine *N*-oxide
Trp	Tryptophan
TRYCATs	Tryptophan catabolites
TUDCA	Tauroursodeoxycholic acid
WT	Wild-type
